# Novel Autophagy-Related Gene Signature Investigation for Patients With Oral Squamous Cell Carcinoma

**DOI:** 10.3389/fgene.2021.673319

**Published:** 2021-06-17

**Authors:** Lihong Huang, Xinghao Yu, Zhou Jiang, Ping Zeng

**Affiliations:** ^1^Department of Biostatistics, Zhongshan Hospital, Fudan University, Shanghai, China; ^2^Department of Biostatistics, School of Public Health, Medical College of Soochow University, Suzhou, China; ^3^Department of Epidemiology and Biostatistics, School of Public Health, Xuzhou Medical University, Xuzhou, China; ^4^Center for Medical Statistics and Data Analysis, School of Public Health, Xuzhou Medical University, Xuzhou, China

**Keywords:** oral squamous cell carcinoma, the cancer genome atlas, autophagy-related gene, prognostic biomarker, cox survival analysis

## Abstract

The correlation between autophagy defects and oral squamous cell carcinoma (OSCC) has been previously studied, but only based on a limited number of autophagy-related genes in cell lines or animal models. The aim of the present study was to analyze differentially expressed autophagy-related genes through The Cancer Genome Atlas (TCGA) database to explore enriched pathways and potential biological function. Based on TCGA database, a signature composed of four autophagy-related genes (*CDKN2A, NKX2-3, NRG3*, and *FADD*) was established by using multivariate Cox regression models and two Gene Expression Omnibus datasets were applied for external validation. Gene Ontology (GO) and Kyoto Encyclopedia of Genes and Genomes (KEGG) pathway enrichment analyses were performed to study the function of autophagy-related genes and their pathways. The most significant GO and KEGG pathways were enriched in several key pathways that were related to the progression of autophagy and OSCC. Furthermore, a prognostic risk score was constructed based on the four genes; patients were then divided into two groups (i.e., high risk and low risk) in terms of the median of risk score. Prognosis of the two groups and results showed that patients at the low-risk group had a much better prognosis than those at the high-risk group, regardless of whether they were in the training datasets or validation datasets. Multivariate Cox regression results indicated that the risk score of the autophagy-related gene signatures could greatly predict the prognosis of patients after controlling for several clinical covariates. The findings of the present study revealed that autophagy-related gene signatures play an important role in OSCC and are potential prognostic biomarkers and therapeutic targets.

## Introduction

Oral cancer is one of the leading causes of death worldwide. Oral squamous cell carcinoma (OSCC) is the most common head and neck squamous cell carcinoma, affecting ~53,000 people and causing around 10,800 deaths in the USA in 2019 (Siegel et al., 2019). The overall 5-year survival rate of OSCC is <60%, and has only slightly improved over the past two decades despite considerable advances in the treatment of OSCC (Vokes et al., 1993; Bagan and Scully, 2008; Scully and Bagan, 2009). Recently, extensive efforts have been devoted to identify molecular prognostic biomarkers for OSCC by integrating DNA methylations and gene expressions (Bai et al., 2013; Yang et al., 2016; Sailer et al., 2017; Shen et al., 2017). Biologically, autophagy is a catabolic process that is involved in the degradation of unimportant or aberrant cellular components through lysosomal hydrolysis, and is a major cellular process that is implicated in an array of cellular and tissue events, including cell stress, endogenous and exogenous cellular component clearance, development, aging, and cancer (Klionsky, 2007; Boya et al., 2013). The cytoprotective role of autophagy serves to prevent cell death under physiological conditions; its self-repair mechanism is exploited by cancer cells to resist therapeutic modalities (Levine and Klionsky, 2004; Mathew et al., 2007). Autophagy is believed to aid in the survival and longevity of cancer cells by buffering metabolic stress, and when conditions permit, can also allow for tumor cell metastases to survive metabolic deprivation and aid in recovery. As inhibiting autophagy in an environment of nutrient deprivation leads to cell death, many current cancer therapies tend to inflict metabolic stress; therefore, autophagy inhibitors may be beneficial for cancer treatment (Yang et al., 2013; Adhauliya et al., 2016).

However, autophagy in tumorigenesis can enhance tumor cell survival under stressful environments, exerting a tumor-promoting effect (Ahn et al., 2011). Some previous studies explored the correlation between autophagy defects and OSCC. For example, G15 (Bai et al., 2013), an antagonist to GPR30, which is a known cancer cell proliferator, and apicidin (Ahn et al., 2011), a histone deacetylase inhibitor, were used to induce autophagy in OSCC. In addition, *PIK3CA* was reported to be frequently mutated in OSCC patients, which resulted in the activation of *PI2K* and downstream effectors, and facilitated autophagy (Sailer et al., 2017). Moreover, high levels of *LC3-II*, which can increase basal levels of autophagy, were revealed to be closely linked to unfavorable OSCC prognosis (Yang et al., 2016).

Although recent studies have demonstrated that autophagy has a complex role in tumorigenesis, drug resistance, and cancer therapy (Kroemer, 2015), relevant studies of OSCC autophagy are still lacking, as most of them only analyzed limited autophagy-related genes in cell lines or animal models (Patil et al., 2015). Based on The Cancer Genome Atlas (TCGA) database (Hoadley et al., 2018), some researchers recently identified 13 autophagy-related genes, but many of these autophagy-related genes cannot be well-validated and had no reasonable biological evidence (Hou et al., 2020). Therefore, it is important to establish a novel autophagy-related gene signature for the prognostic prediction of OSCC patients to explore the potential biological function of autophagy-related genes. In the present study, we aimed to identify differentially expressed autophagy-related genes based on TCGA, and established a signature composed of 4 autophagy-related genes using the multivariate Cox regression model. Two Gene Expression Omnibus (GEO) datasets (GSE85446 and GSE41613) was applied to validate the performance of the constructed autophagy-related gene signature. Furthermore, Gene Ontology (GO) and Kyoto Encyclopedia of Genes and Genomes (KEGG) pathway enrichment analyses were performed to explore the function of these autophagy-related genes and their pathways. The findings of the present study indicated that autophagy-related gene signatures play an important role in the survival risk of OSCC patients and might serve as potential prognostic biomarkers and therapeutic targets.

## Materials and Methods

### Patient Samples of OSCC in TCGA and GSE85446

We downloaded normalized messenger RNA (mRNA) expression profiles of 660 OSCC samples from TCGA (Hoadley et al., 2018). After quality control, a total of 502 tumor and 44 normal samples were reserved to identify differentially expressed genes (DEGs), of which autophagy-related genes were of particular interest. Furthermore, after excluding samples with missing survival time and normal samples, 499 tumor samples were kept for establishing the risk score pattern based on these autophagy-related DEGs and performing a correlation analysis for survival and clinical features. Based on prior studies Shen et al. (2017, 2018) and Yu et al. (2020), and following the suggestion given in Liu et al. (2018), we only considered the overall survival time in the present study as there was minimal ambiguity in defining an overall survival event. In brief, overall survival in TCGA was the duration from diagnosis to death. The median overall survival time of TCGA OSCC patients was 639 days, with the censoring rate being 56.5%. We also evaluated the prediction performance of the constructed risk score using two external GEO datasets (accession ID: GSE85446 and GSE41613). The detailed information of these datasets after quality control is summarized in Table 1.

**Table 1:** Detailed information of the training and validation datasets.

**Features**		**TCGA**	**GSE85446**	**GSE41613**
Censor (*n*, %)		277 (55.5)	34 (51.5)	34 (51.5)
Age (mean ± SD)		61.0 ± 12.0	60.7 ± 9.6	59.07^#^
Gender (*n*, %)	Male	410 (73.2)	39 (59.1)	66 (68.0)
	Female	150 (26.7)	27 (40.9)	31 (32.0)
Clinical stage (*n*, %)	Advanced (III-IV)	413 (73.8)	46 (69.7)	56 (57.7)
	Early (I-II)	133 (23.8)	20 (30.3)	41 (42.3)
	NA	14 (2.5)	0 (0)	0 (0)
Tobacco smoking history (*n*, %)	Current/former	418 (74.6)	/	/
	Never	127 (22.7)	/	/
Race (*n*, %)	White	483 (86.3)	/	/
	Other	62 (11.1)	/	/

# *Age was a categorical variable in GSE41613, so the standard deviation cannot be calculated*.

### Identification of DEGs

The R limma package (version 3.46.0) was applied to detect genes that were differentially expressed between normal and tumor samples in TCGA OSCC dataset (Ritchie et al., 2015). After normalization and gene ID alignment, the expression matrix of the OSCC cancer data set was employed as an input file. A linear model was used to calculate the coefficients and the standard errors. Empirical Bayesian conditioning was then used to narrow the standard errors that were much larger or smaller compared to the mean from the other genes. Following prior studies (Korbolina et al., 2014; Caputo et al., 2020), the screening criteria for differential expression were set as |log_2_FC| > 1.0 with a false discovery rate (FDR) of <0.05 in our analyis.

### Functional and Pathway Enrichment Analyses

We referred to DEGs as autophagy-related genes by matching them with genes that had been found to attend in the autophagy regulatory process in the Human Autophagy Database (HADb; http://autophagy.lu/clustering/index.html) (Homma et al., 2011). We identified a total of 24 autophagy-related DEGs in TCGA OSCC dataset. Based on these autophagy-related genes, we conducted GO and KEGG pathway enrichment analyses with the R clusterProfiler package (version 3.18.0) (Zou et al., 2020). In brief, GO database annotates gene products from molecular functions, biological processes, and cellular components of biology, while KEGG pathway analysis identifies DEG pathway enrichment, which can facilitate further mechanism research. We also constructed a network diagram to demonstrate the relationship between GO terms and differential genes and gene overlap relationships between enriched pathways (Zou et al., 2020). In the network diagram plot, each cluster represents an enriched pathway; the top 30 enriched pathways were drawn by default, with the size of the node corresponding to the number of differentially enriched genes in that pathway.

### Risk Score Establishment

Based on TCGA OSCC tumor samples (*n* = 499), the 24 autophagy-related genes were first analyzed using the univariate Cox regression, with age, race, sex, and clinical stage as covariates. Four genes, which were significant in terms of the univariate regression, were further referred to as autophagy-related prognostic signatures. Next, multivariate Cox regression was performed to evaluate the risk score of the 4 genes for the overall survival of OSCC patients. Cox regressions were conducted under the proportional hazards (PH) assumption in terms of the method proposed in Grambsch and Therneau (1994). The risk score could be obtained for each patient using the product of the gene expression and estimated coefficients from the multivariate model (Luan et al., 2019; Yu et al., 2019, 2020; Gao et al., 2020). The patients were then divided into two groups (low risk and high risk) according to the median of risk score across all individuals. The Kaplan-Meier curve and the log-rank test were used to assess the prognostic capability of the 4-gene signature in TCGA or GSE85446/GSE41613 OSCC patients. Finally, because the status of cancer and the associated genes change over time in practice, we calculated the inverse probability of censoring weighting estimation. A time-dependent receiver operating characteristic (ROC) curve was performed to evaluate the survival of patients using the nearest neighbor method (Uno et al., 2007; Blanche et al., 2013).

**Figure 1:**
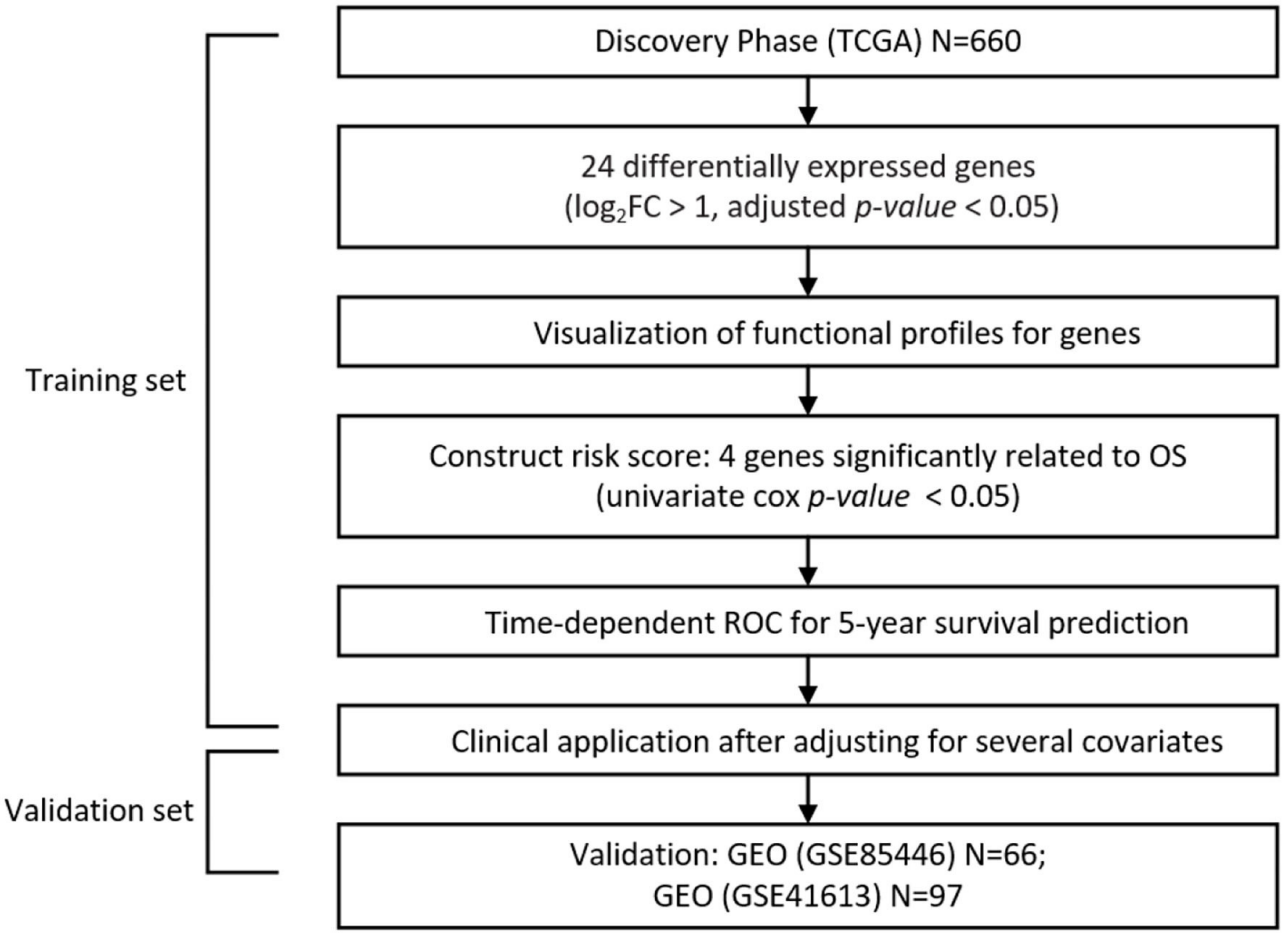
Flow chart indicating the study design of the present work.

The workflow of our analysis is presented in Figure 1. All graphs and statistical analyses were conducted by R software (version 3.5.1, The R Foundation for Statistical Computing) (Ihaka and Gentleman, 1996). The tests in our study were 2-tailed, and a *P*-value or FDR <0.05 was regarded as the cutoff value for statistical significance.

## Results

### Differential Expression Analysis and Autophagy-Related Genes

**Figure 2:**
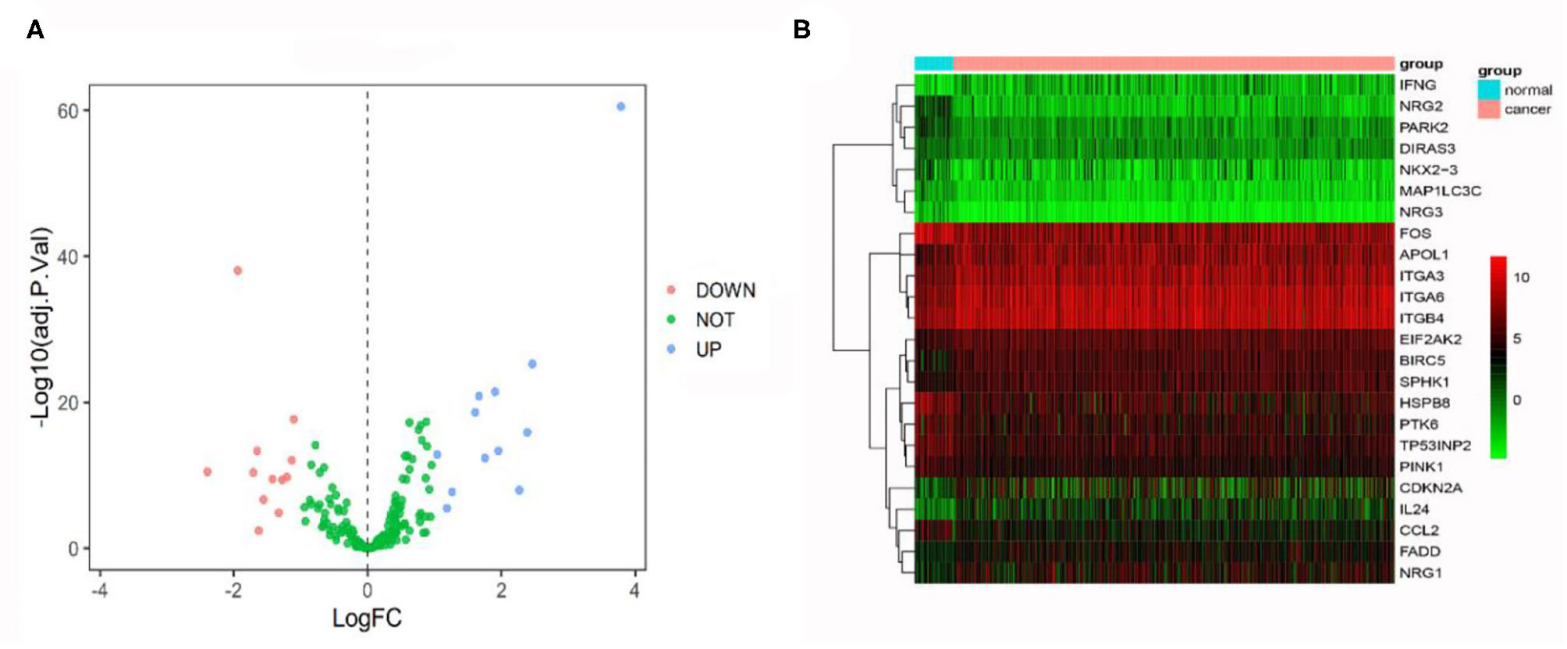
**(A)** Volcano plot comparing autophagy-related gene expression for tumor and non-tumor tissues. A total of 24 genes were identified [red (down-regulated) and blue points (up-regulated)]. **(B)** Heatmap showing 24 genes in tumor tissues and adjacent non-tumor tissues.

First, we examined DEGs in TCGA OSCC patients based on 502 normal and 44 tumor samples. A volcanic plot was created to illustrate the significance and reliability of differential expression between the two groups (Figure 2A). We identified a total of 4,409 DEGs with FDR <0.05 and |log_2_FC| > 1. After screening the HADb database, we identified 24 autophagy-related genes (e.g., *NRG2, BIRC5*, and *PARK2*) for further analysis (Figure 2B). Of these autophagy-related genes, 12 were up-regulated (log_2_FC > 1), while the rest were down-regulated (log_2_FC < −1) (Table 2).

**Table 2:** A total of 24 autophagy-related genes identified in TCGA data set.

**Gene**	**log_**2**_FC**	**Regulate**	**Average**	***t***	**Adjust *P***
*NRG2*	3.777	Up	−2.150	19.601	4.88E-61
*PARK2*	2.464	Up	−0.877	11.651	5.03E-26
*MAP1LC3C*	1.897	Up	−2.951	10.586	4.83E-22
*NRG3*	1.664	Up	−3.902	10.458	1.42E-21
*TP53INP2*	1.603	Up	5.019	9.807	2.92E-19
*HSPB8*	2.383	Up	5.134	9.015	1.37E-16
*FOS*	1.946	Up	7.660	8.198	5.19E-14
*PINK1*	1.032	Up	4.313	8.028	1.68E-13
*CCL2*	1.740	Up	3.344	7.873	4.85E-13
*NKX2-3*	2.271	Up	−2.300	6.290	9.07E-9
*DIRAS3*	1.250	Up	−0.740	6.161	1.84E-8
*PTK6*	1.178	Up	4.704	5.149	3.24E-6
*BIRC5*	−1.944	Down	5.322	−14.706	1.16E-38
*EIF2AK2*	−1.110	Down	5.921	−9.646	1.05E-18
*ITGA6*	−1.661	Down	8.703	−8.273	3.08E-14
*SPHK1*	−1.141	Down	5.333	−7.801	7.90E-13
*IL24*	−2.415	Down	2.109	−7.349	1.55E-11
*APOL1*	−1.715	Down	7.324	−7.213	3.70E-11
*ITGB4*	−1.210	Down	8.911	−6.969	1.69E-10
*ITGA3*	−1.433	Down	7.996	−6.879	2.93E-10
*FADD*	−1.284	Down	3.631	−6.833	3.87E-10
*NRG1*	−1.570	Down	4.054	−5.765	1.55E-7
*IFNG*	−1.331	Down	−2.047	−4.852	1.27E-5
*CDKN2A*	−1.629	Down	2.712	−3.346	3.90E-3

### Pathway Enrichment Analysis

**Figure 3:**
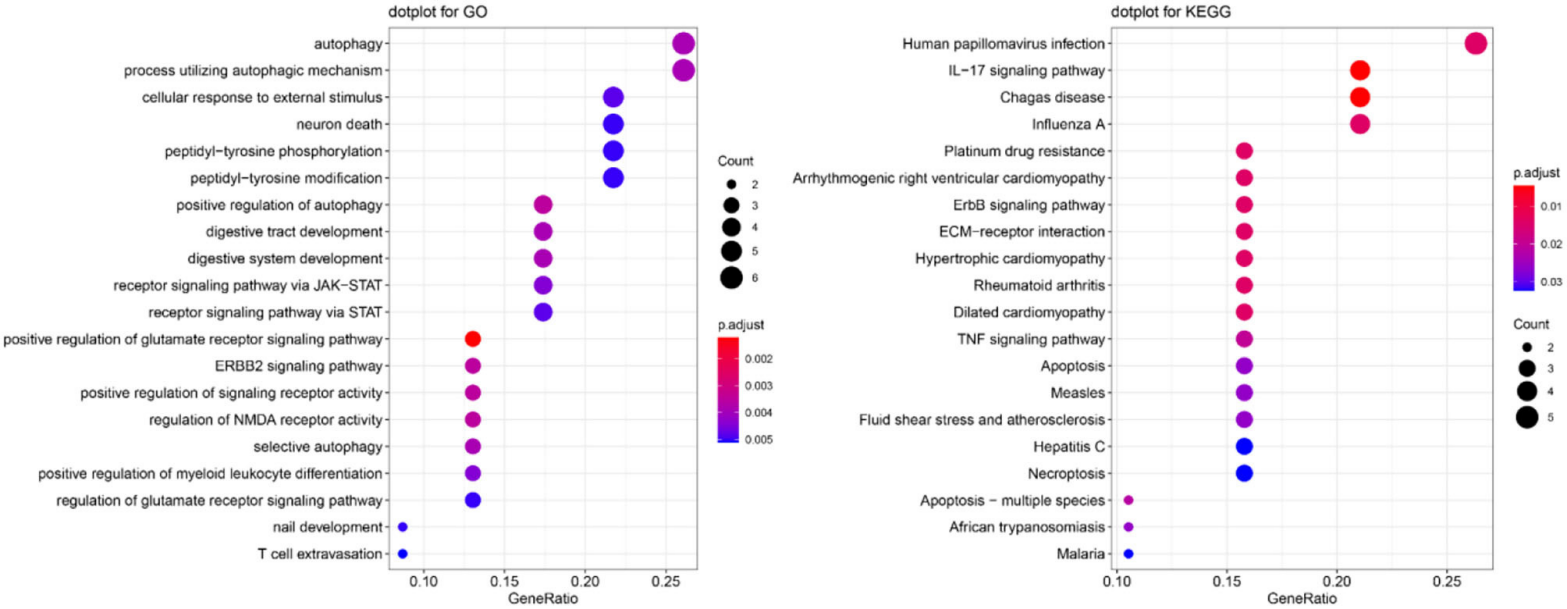
GO and KEGG pathway enrichment analyses for 24 autophagy-related genes.

To explore the function of these autophagy-related genes and their pathways, we performed GO and KEGG enrichment analyses. The most significant GO and KEGG pathways are presented in Figure 3. We found that these genes were enriched in several key pathways related to autophagy or OSCC (e.g., autophagy, process utilizing autophagic mechanism, and human papillomavirus infection). For BP, positive regulation of the glutamate receptor signaling pathway, ERBB2 signaling pathway, positive regulation of signaling receptor activity, regulation of N-methyl-D-aspartate receptor (NMDA) receptor activity, and positive regulation of autophagy were the most enriched categories. For CC ontology, enriched categories included the integrin complex and protein complex involved in cell adhesion. For MF, receptor ligand activity, signaling receptor activator activity, insulin-like growth factor I binding, and cytokine activity were commonly enriched. The gene-concept network depicts the linkages of autophagy-related genes and biological concepts (i.e., GO terms or KEGG pathways) as a network (Supplementary Figure 1), while the enrichment plot that enriched terms into a network with edges connecting overlapping gene sets shows the clusters of several related genes (Supplementary Figure 2).

### Genetic Risk Score Analysis

**Figure 4:**
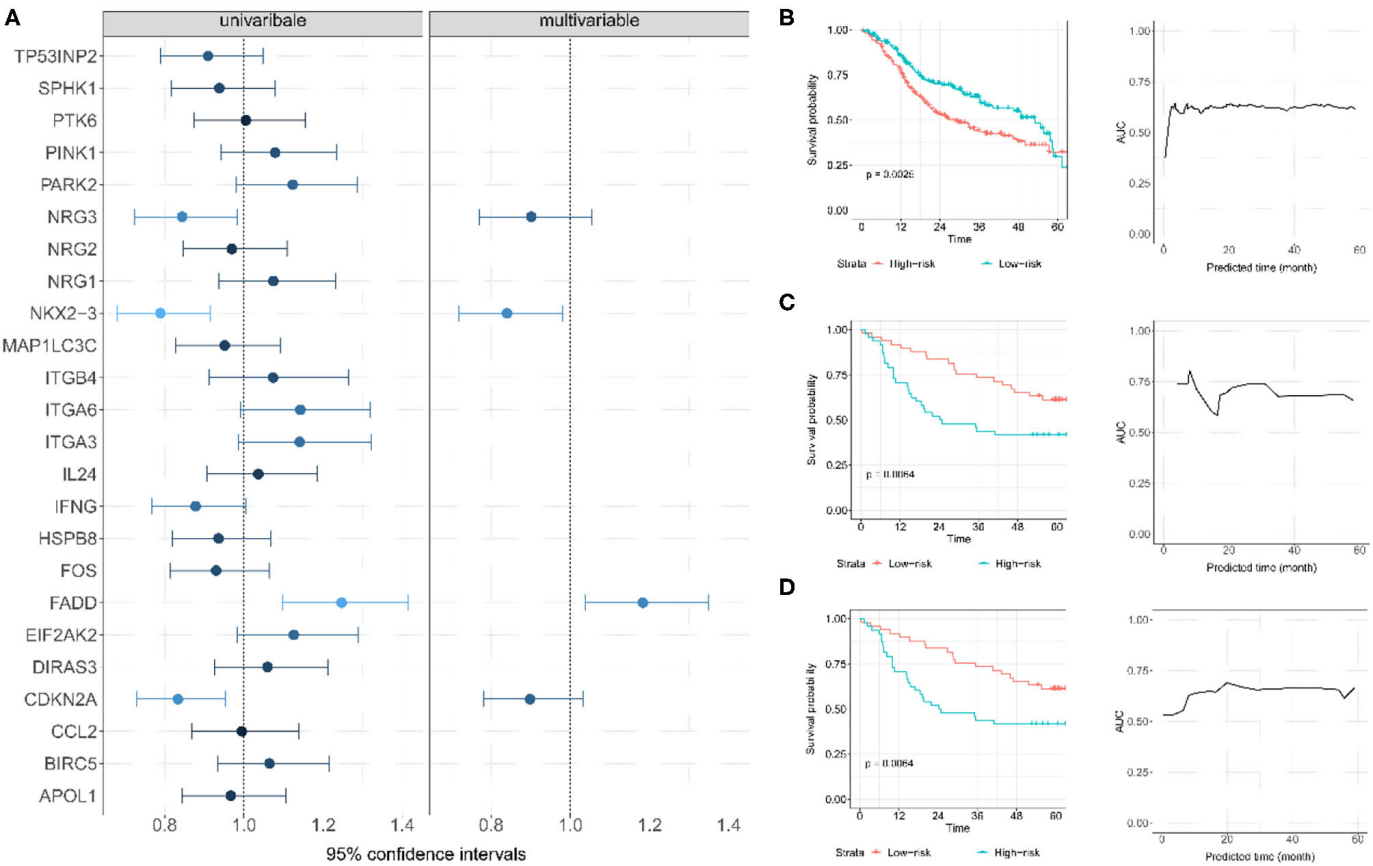
**(A)** Establishment of autophagy-related gene signature and predictive value analysis for OS of OSCC patients based on TCGA dataset. **(B)** The survival probability and time-dependent ROC curve for TCGA; each patient was divided into low- and high-risk score groups according to the median of the risk score; **(C)** The survival probability and time-dependent ROC curve for GSE85446. **(D)** The survival probability and time-dependent ROC curve for GSE41613.

The Kaplan-Meier survival analysis was utilized to evaluate the relationship between each autophagy-related gene and the overall survival time in TCGA OSCC data set (Supplementary Figure 3). Four genes (*CDKN2A, NKX2-3, NRG3*, and *FADD*) were significantly associated with overall survival. Subsequently, these genes were treated as prognosis-related genes for further analysis. Using the multivariate Cox model with TCGA OSCC as the training data, we constructed a prognostic risk score with these four genes as follows:−0.174 × *NKX2-3*-0.104 × *NRG3*+0.168 × *FADD*-0.108 × *CDKN2A* (Figure 4A). Patients were then divided into low- and high-risk score groups according to the median of the risk score (median = 0.031). Compared with patients in the low-risk score group, patients in the high-risk score group had a substantially shorter survival (the median overall survival was 58.7 months in the low-risk score group and 36.0 months in the high-risk score group; *P* = 1.34E-05) (Figure 4B). A time-dependent ROC curve indicated that the 4-gene based prognostic model had meaningful predictive accuracy, with an average of the area under the curve (AUC) being 0.615 (range 0.375–0.644) across the survival time (Figure 4B).

**Figure 5:**
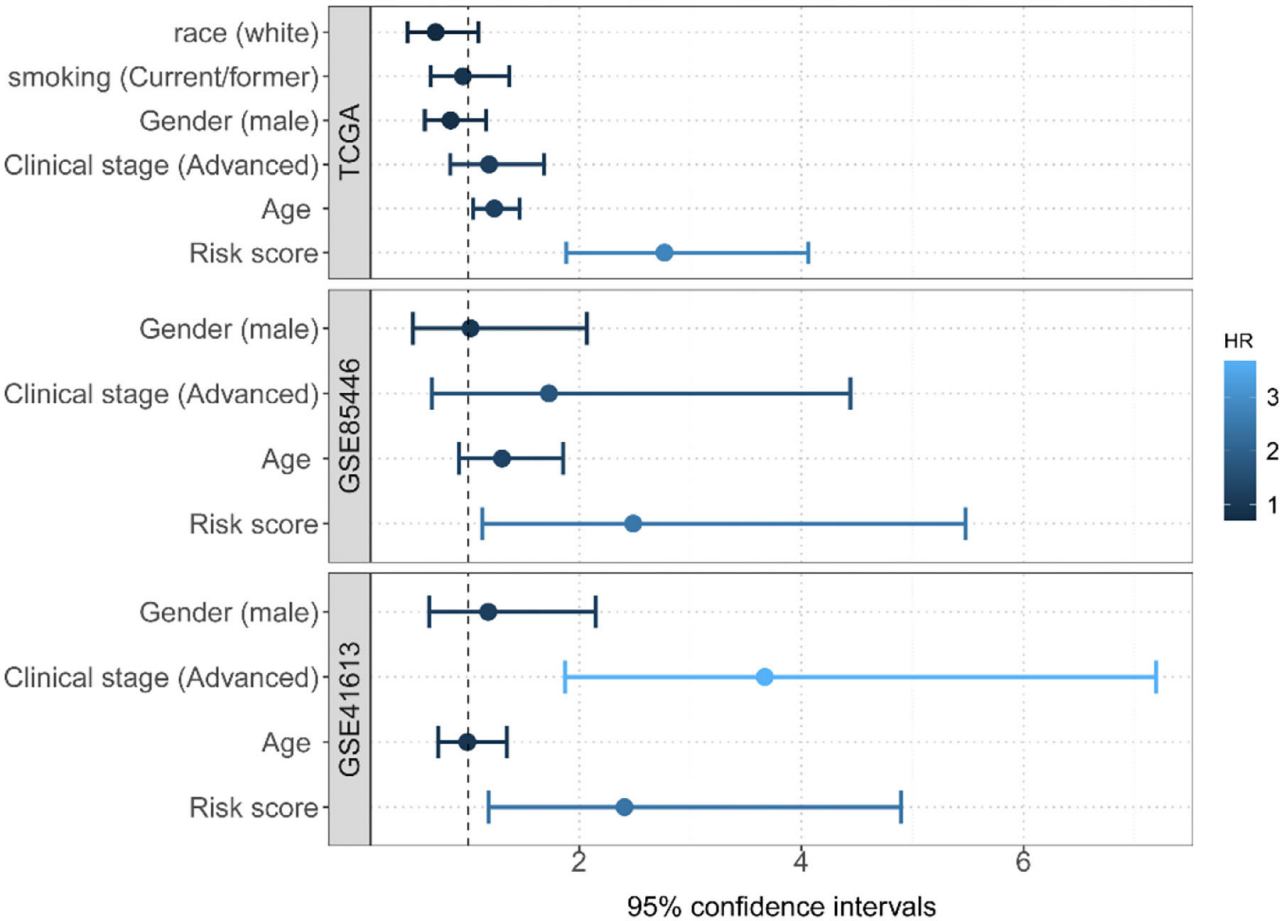
Multivariate Cox regression analyses of the risk score constructed by four autophagy-related gene signatures and predictive clinic pathological factors of overall survival (OS) based on TCGA and two GEO datasets.

Two GEO datasets (i.e., GSE41613 and GSE85446) were further applied as the test data for evaluating the prognostic performance of the risk score. The risk score for each patient in this dataset was first calculated. Based on its median, these patients were then divided into two groups (high risk or low risk). Of note, the median overall survival time was 84.1/65.0 months and the censoring rate was 51.5/47.4% for GSE85446 and GSE41613, respectively. Patients with a low autophagy-related risk score were found to have a higher survival probability compared with those with a high-risk score in both GSE85446 (*P* = 0.024) (Figure 4C) and GSE41613 (*P* = 0.021) (Figure 4D). The time-dependent ROC indicated that the 4-gene-based prognostic model had meaningful predictive accuracy, with an average AUC of 0.689 (range 0.584–0.805) across the survival time for GSE85446 (Figure 4C) and an average AUC of 0.648 (range 0.533–0.724) across the survival time for GSE41613 (Figure 4D). Moreover, after adjusting for other available covariates (e.g., sex), the Cox multivariate regression indicated that the risk rate of the risk score was 2.73 (95% confidence intervals [CIs] 1.74–4.28; *P* = 1.34E-5) in TCGA, 2.49 (95% CIs 1.13–5.48; *P* = 0.024) in GSE85446, and 2.36 (95% CIs 1.14–4.92; *P* = 0.021) in GSE41613 (Figure 5), indicating that a higher autophagy-related risk score led to a reduced chance of prolonged survival. This finding also indicated that the autophagy-related risk score was an independent predictor of patient prognosis. Moreover, we performed a sensitive analysis by removing some patients who had very short survival time (<14 days). The results were consistent with these shown here, although an additional gene (*ITGA6*) was identified (Supplementary Material).

### Four Autophagy-Related Genes

According to the multivariate Cox model, our results revealed that among the 4 autophagy-related genes, *CDKN2A, NKX2-3*, and *NRG3* were protective, while *FADD* was associated with the survival risk (Figure 4A). *CDKN2A* is known as cyclin-dependent kinase inhibitor 2A. It is a gene located on chromosome 9p21 and has 3 exons which encode for tumor-suppressor protein p16 (Soria et al., 2001; Burke et al., 2013; Lim et al., 2014). *CDKN2A* has been found to be inactivated in a broad spectrum of solid tumors and in more than 80% of OSCC (Nielsen et al., 1998; Prigge et al., 2015; Pal et al., 2016). The low expression of *CDKN2A* was significantly associated with recurrence in OSCC patients, and the overall survival in patients decreased in patients with a reduction of *CDKN2A* expression (Padhi et al., 2017), which was consistent with our results that *CDKN2A* is a potential favorable gene in OSCC.

*NKX2-3* is a member of the homeobox NKX family. *NKX2* homeobox family proteins are well-known for their crucial role in cancer development and progression. *NKX2-3* was verified to stimulate the activation of B-cell receptor signaling and drive carcinogenesis through triggering the NF-kB and PI3KAKT pathways (Robles et al., 2016). The *NKX2-3* protein was found to be located in the nucleus of tumor cells and was classified as a promising biomarker to predict the response of cancer patients undergoing *FOLFOX4* chemotherapy (Li et al., 2012). *NKX2-3* has also been identified as a prognostic signature for head and neck squamous cell carcinoma in previously published studies (Jin and Qin, 2020; Zhu et al., 2020).

The *NRG3* gene at 10q22-q24 has been implicated in multiple psychiatric traits, such as cognitive impairment. It has been reported that *NRG3* is associated with the risk and age at onset of Alzheimer's disease (Wang et al., 2014). *NRG3* was recently identified as a prognostic index for inpatients with head and neck squamous cell carcinoma (Feng et al., 2020).

*FADD* is a gene located on the 11q13.3 region of chromosome 11 in humans and encodes Fas-associated protein with death domain, also called *MORT1* (Kim et al., 1996). Previous studies have found that *FADD* plays an important role in cell growth and cell proliferation (Hueber et al., 2000). The results from a number of studies of human malignancies have revealed the highly controversial relationship between *FADD* expression and cancer progression (Tomioka et al., 2006; Gibcus et al., 2007). It was reported that the expression of *FADD* was higher when compared with that of adjacent areas, which might be determined car genomic amplification in llql3.3 in OSCC, OSCC cells expressing *FADD* are more likely to metastasize and lead to poor survival rates (Prapinjumrune et al., 2010). This conclusion is consistent with our own findings that showed *FADD* was an unfavorable survival predictor for OSCC patients.

## Discussion

It has been previously reported that autophagy has complex roles in tumorigenesis, drug resistance, and cancer therapy (Kroemer, 2015). Studies on OSCC autophagy are limited, and much remains to be explored about the role autophagy playing in the progression of head and neck cancers and its use in cancer therapy (Patil et al., 2015). In the present study, we discovered and described the differential expression profiles of 24 autophagy-related genes in OSCC. Based on biological function analysis and pathway enrichment of DEGs, we found that these genes were mainly enriched in key pathways, such as autophagy, the ERBB2 signaling pathway, and process utilizing autophagic mechanism, indicating that 24 autophagy-associated DEGs may be involved in the development, progression, and drug resistance of head and neck cancer.

Through a network diagram, we also found that 8 of the 24 autophagy-related genes (*TP53INP2, HSPB8, PTK6, NRG2, NRG1, PINK1, IFNG*, and *CCL2*) were involved in the regulation of the following multiple pathways: positive regulation of glutamate receptor signaling, ERBB2 signaling, positive regulation of signaling receptor activity, regulation of NMDA receptor activity, and positive regulation of autophagy These pathways are potentially associated with OSCC development and metastasis. Previously published studies have shown that glutamate is a potential growth factor for tumor development; for example, glioma cells produce glutamate *in vivo* in neurotoxic amounts (Takano et al., 2001). ERBB2 receptors are involved in a variety of important functions in organisms controlled by members of the ERBB receptor family, including cell growth, differentiation, and apoptosis. Simultaneous activation of the ERBB2 receptor signaling pathway can enhance various properties associated with metastasis, leading to an increase in cancer metastasis (Yu and Hung, 2000). Activation or antagonism of NMDA receptors may be associated with anti-proliferative and anti-invasive effects, which can affect the proliferation rate of a wide range of cell lines in a variety of cancers. In addition, targeting NMDA receptors expressed on the surface of cancer cells can be used as a therapeutic strategy for cancer (Deutsch et al., 2014).

Furthermore, we constructed an autophagy-related gene signature and divided OSCC patents into high- and low-risk groups. The multivariate Cox regression model showed that the autophagy-related gene signature was a significant prognostic factor for OSCC, and survival analysis demonstrated that patients in the high-risk group have significantly shorter median survival time compared with those in the low-risk group, which was validated in another GEO datasets. Additionally, of the 4 identified autophagy-related genes, three were potential favorable genes (*CDKN2A, NKX2-3*, and *NRG3*) and 1 was a potential unfavorable gene (*FADD*). These four autophagy-related genes have been reported to be closely associated with the development and prognosis of OSCC or other malignancies. Besides, *ITGA6* was also identified in our sensitive analysis. As a transmembrane glycoprotein adhesion receptor protein, *ITGA6* is widely upregulated in many types of tumors and plays a role in migration and invasion of cancer cell. Further investigations of these autophagy-related genes in OSCC tissues, cell lines, and animal models are recommended to clarify the potential molecular mechanisms of these autophagy-related genes in impacting survival outcomes of OSCC patients.

Based on public databases, our study comprehensively explored and verified the prognostic usefulness of an autophagy-related gene signature in different OSCC subgroups. The findings of the present study could be employed as a reference for follow-up functional investigations of autophagy-related genes in OSCC and targeting drug development. However, our work still had several limitations. First, the autophagy-related gene signature only showed moderate prediction ability in our study; thus, it might be not a powerful indicator to predict the prognosis of OSCC patients. Further studies integrating multi-omics datasets might improve the predictive performance (Zhao et al., 2014). Second, the censored rate in our analyzed datasets was relatively high, which might undermine the power of the survival analysis, leading to the failure of identifying more autophagy-related genes.

## Conclusion

We identified and confirmed a novel four autophagy-related genes signature for OSCC patients. Our work also provided meaningful signature for the molecular mechanism study of autophagy-related genes in OSCC.

## Data Availability Statement

The datasets presented in this study can be found in online repositories. The names of the repository/repositories and accession number(s) can be found in the article/Supplementary Material.

## Author Contributions

PZ and LH conceived the idea for the study. LH and XY obtained the data and performed the data analyses. LH, ZJ, and XY cleared up the datasets. PZ, LH, and XY interpreted the results of the data analyses and drafted the manuscript. All authors approved the manuscript and provided relevant suggestions.

## Conflict of Interest

The authors declare that the research was conducted in the absence of any commercial or financial relationships that could be construed as a potential conflict of interest.
