# Supplementary material for: Novel Autophagy-Related Gene Signature Investigation for Patients With Oral Squamous Cell Carcinoma
Source: Front Genet. 2021 Jun 17;12:673319. doi: 10.3389/fgene.2021.673319 (PMC8248343; doi:10.3389/fgene.2021.673319)
Supplement: Supplementary file 1 [file Data_Sheet_1.docx]

# Supplemental File

*Sensitive analysis*

To study the influence of short survival time, we removed four patients whose OS time < 14 days from our Cox model. As a result, five genes (*ITGA6*, *CDKN2A*, *NKX2-3*, *NRG3*, and *FADD*) were significantly associated with overall survival. Subsequently, these genes were treated as autophagy-related genes for further analysis. Using the multivariate Cox model, we constructed a prognostic risk score with these 5 genes as follows

0.043×*ITGA6*-0.171×*NKX2-3*-0.104×*NRG3*+0.168×*FADD*-0.108×*CDKN2A*

Compared with patients in the low-risk score group, patients in the high-risk score group had a substantially shorter survival (*P*=5.37E-6). A time-dependent ROC curve indicated that the 5-gene based prognostic model had satisfactory predictive accuracy, with an average of the area under the curve (AUC) being 0.631 (range 0.485-0.771) across the survival time. Patients with a low autophagy-related risk score were found to have a higher survival probability compared with those with a high-risk score in GSE85446 (*P*=0.010) and GSE41613 (*P*=0.015). Moreover, after adjusting for other available covariates (e.g., sex), Cox multivariate regression indicated that the risk rate of the risk score was 2.77 (95% confidence intervals [CIs] 1.77-4.22; *P*=5.37E-6) in TCGA, 2.55 (95% CIs 1.25-5.20; *P*=0.010) in GSE85446 and 2.55 (95% CIs 1.25-5.20; *P*=0.010) in GSE41613, indicating that a higher autophagy-related risk score leads to a reduced chance of prolonged survival.


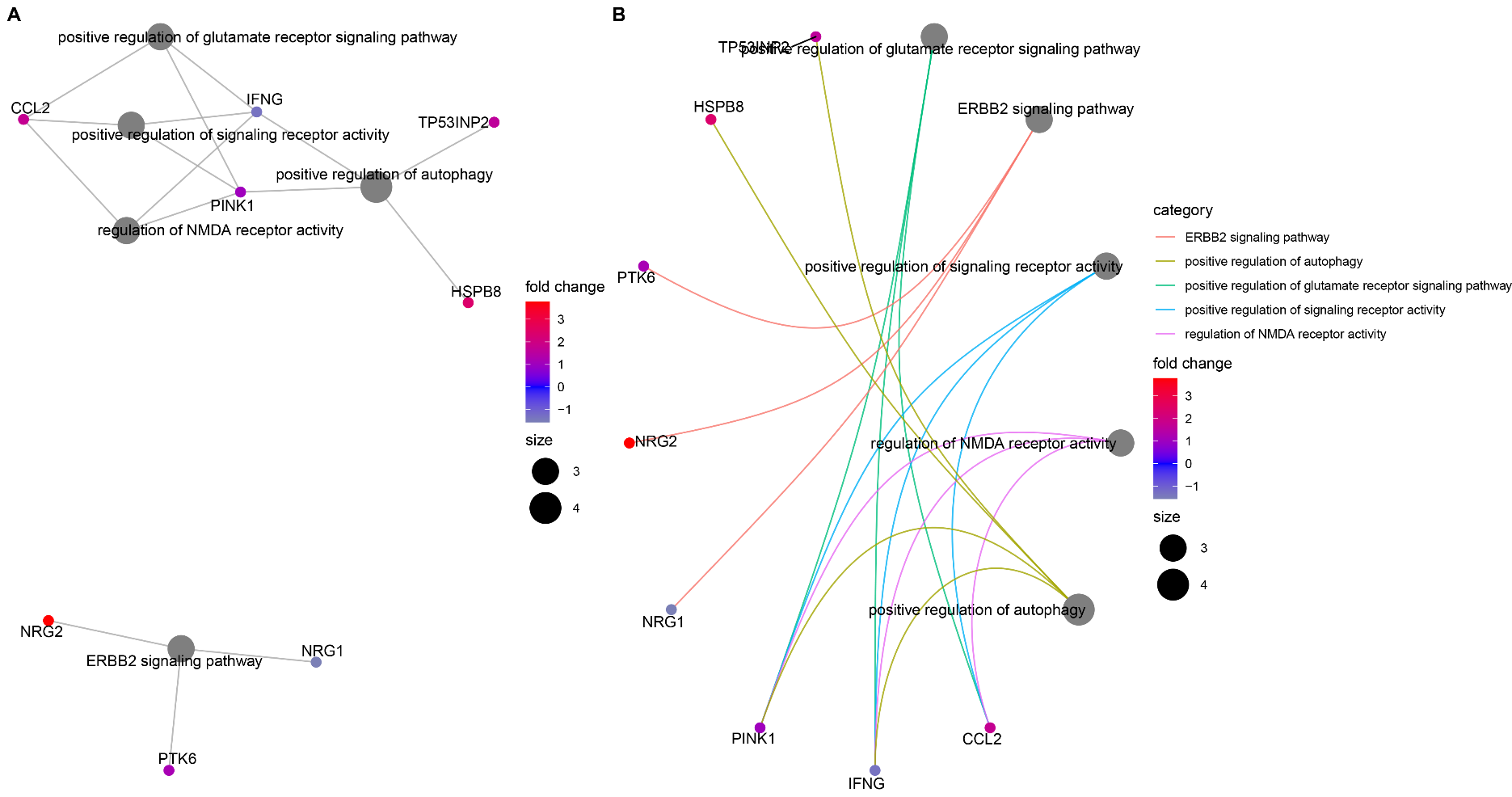


Figure S1. Gene-concept network depicting the linkages of autophagy-related genes and biological concepts (i.e., GO terms or KEGG pathways) as a network.


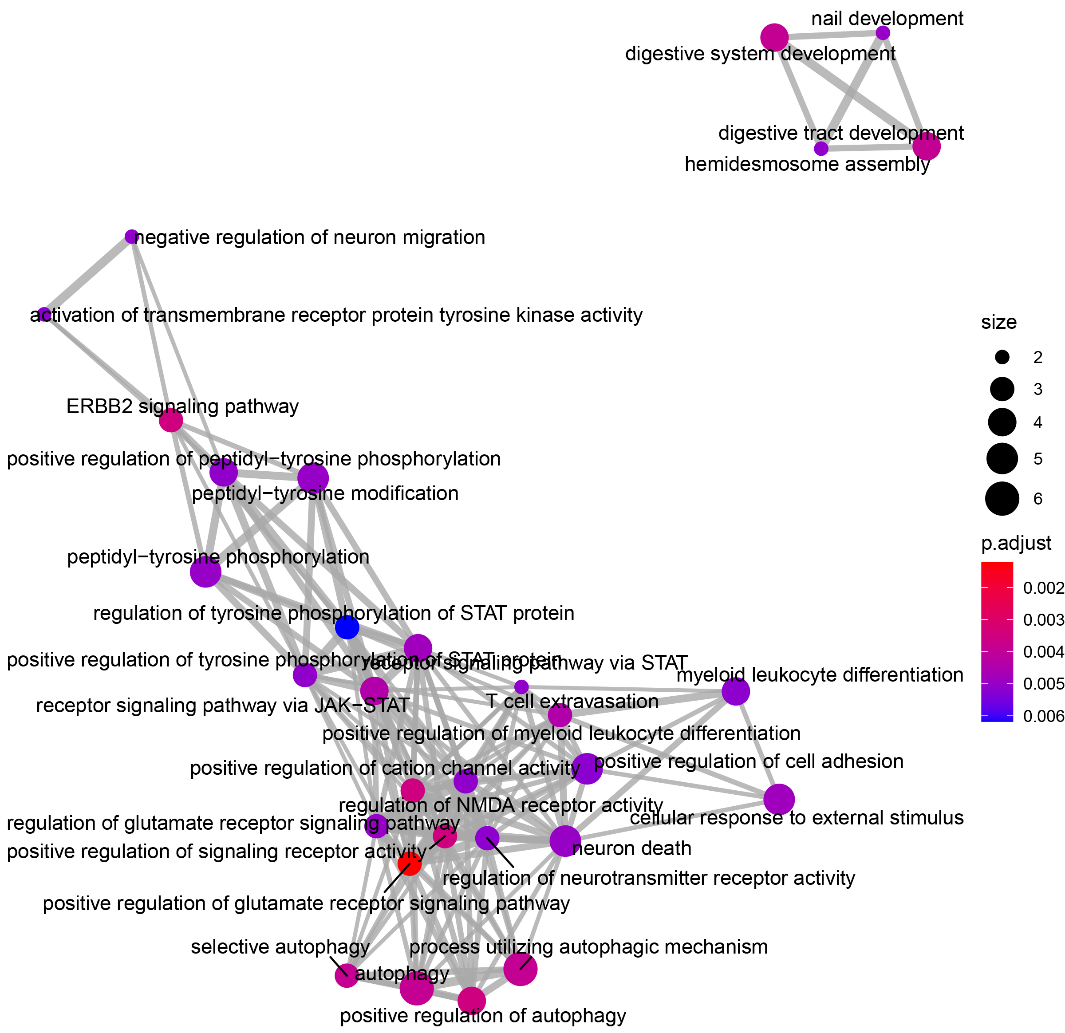


Figure S2. Enrichment plot that enriched terms into a network with edges connecting overlapping gene sets showing the clusters of several related genes.


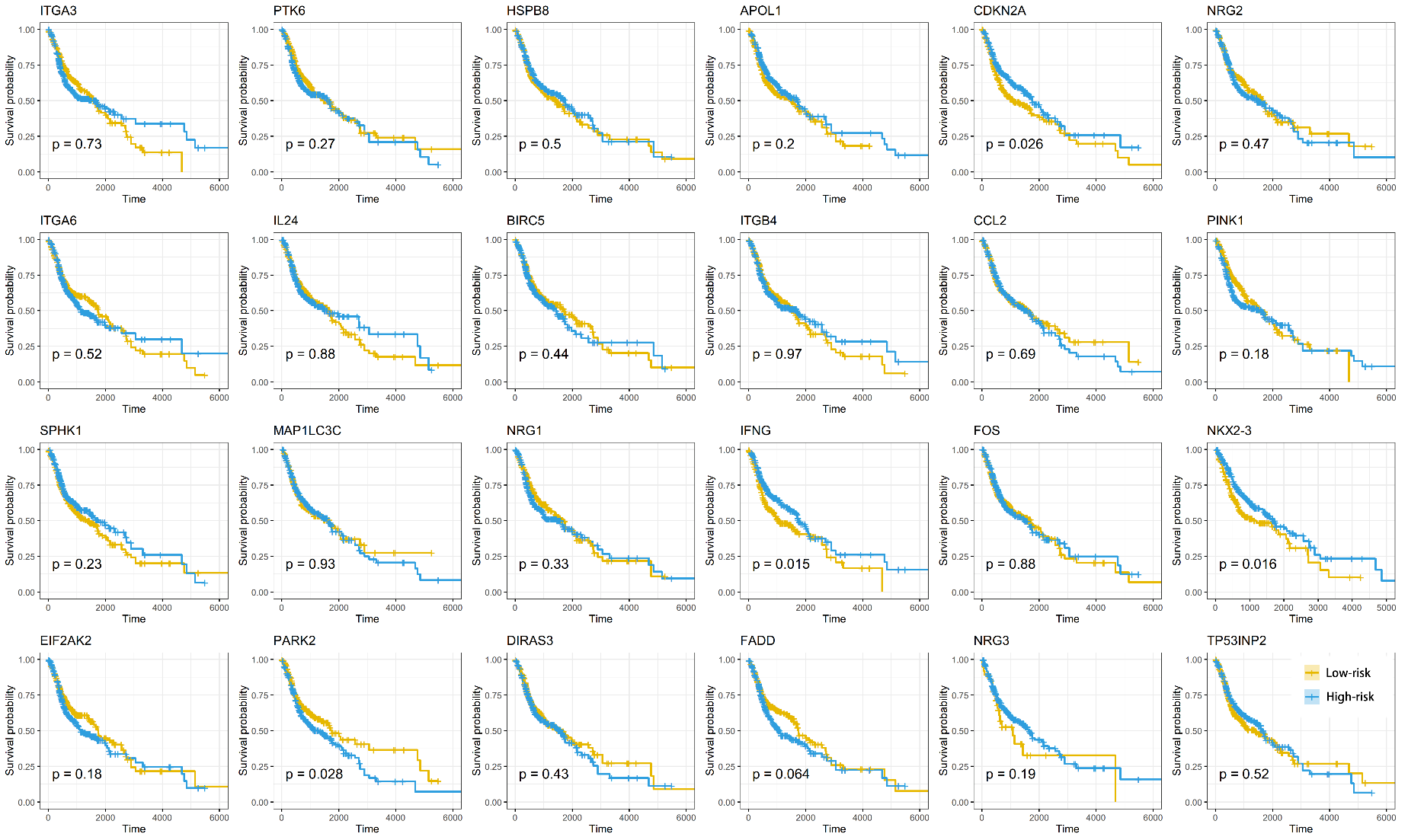


Figure S3. Survival plot for each gene in TCGA OSCC data set. Each patient was divided into low- and high-risk score groups according to the median of the risk score.
